# A Self-Healing Composite Film Made of Cellulose Nanocrystals and a Polyvinyl Acetate Copolymer

**DOI:** 10.1021/acsapm.5c00219

**Published:** 2025-04-16

**Authors:** Guofan Xu, Jude Laverock, Todor T. Koev, Yaroslav Z. Khimyak, Onajite Abafe Diejomaoh, Sebastien Rochat, Stephen J. Eichhorn

**Affiliations:** 1 Bristol Composites Institute, School of Civil, Aerospace and Design Engineering, University of Bristol, University Walk, Bristol BS8 1TR, U.K.; 2 School of Chemistry, University of Bristol, Bristol BS8 1TS, U.K.; 3 School of Chemistry, Pharmacy and Pharmacology, University of East Anglia, Norwich Research Park, Norwich NR4 7TJ, U.K.; 4 School of Engineering Mathematics and Technology, University of Bristol, Bristol BS8 1TW, U.K.

**Keywords:** cellulose nanocrystals, polyvinyl acetate, AGET ATRP, macroinitiator, self-healing

## Abstract

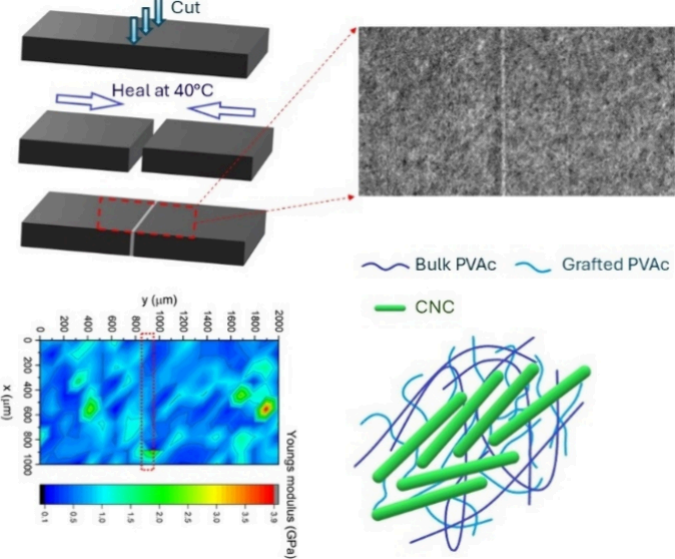

A cellulose nanocrystal (CNC)-polyvinyl acetate (PVAc) self-healing composite film was fabricated using a grafting-from approach generating polyvinyl acetate (PVAc) chains on CNC macroinitiators. These grafted-to CNCs were then mixed with bulk PVAc polymer to form a composite. Fourier-transform infrared spectroscopy, X-ray photoelectron spectroscopy, and solid-state nuclear magnetic resonance were used to demonstrate the presence of the grafted PVAc chains on the surface of the CNCs. Transmission electron microscopy images revealed the structure of the modified CNCs, which formed closely packed clusters due to the grafted PVAc chains. The thermal properties of the CNCs and their composite films were assessed by using differential scanning calorimetry, determining the appropriate temperature for the healing of the composite film. On this basis, the film was cut into two pieces and rejoined and healed in an oven heated at 40 °C for 6 h. The healed sample was viewed under an optical microscope and electron microscopy, demonstrating the efficacy of the healing process. An array of microindentation tests across the surface of the healed specimen was conducted to quantify stiffness, revealing no detectable differences between the healed and intact regions. This healing was found to only occur for the grafted-to samples and was not evident for the composites made of PVAc and ungrafted CNCs. This work demonstrates that grafting polymer chains onto CNCs and blending these with a bulk polymer are promising approaches for fabricating composite films capable of healing macroscopic fractures.

## Introduction

Polyvinyl acetate (PVAc), as a biocompatible synthetic thermoplastic, has been investigated for the fabrication of coatings, hydrogels, adhesives, and films.^1^ PVAc is a synthetic oil-based polymer, normally produced through free radical polymerization of the monomer vinyl acetate.^2−5^ Controlled/living radical polymerization (CRP) of vinyl acetate to obtain polymers with well-defined molecular weight distributions has been achieved using a number of approaches, including metal-mediated CRP with triisobutylaluminum,^6−8^ reversible addition–fragmentation transfer polymerization (RAFT) or macromolecular design via an interchange of xanthates (MADIX) process with dithiocarbamates and xanthates,^9−12^ iodine degenerated transfer poymerisation,^13,14^ and transition metal-mediated atom transfer radical polymerization (ATRP) with iron^15,16^ and copper^17−19^ complexes.

PVAc has been applied in self-healing composite materials by blending with other polymers.^20−22^ A poly(vinyl alcohol)–poly(ethylene glycol) graft copolymer has been blended with PVAc and coated on chlorpheniramine maleate pellets exhibiting high robustness, compressibility, and storage safety.^20^ A swelling-based self-healing was demonstrated in the film after being damaged with a needle.^20^ A PVAc and poly(ε-caprolactone) (PCL) blend has been fabricated into an interwoven polymeric composite, yielding self-healing and shape memory functionalities through dual-electrospinning.^21^ This PVAc/PCL (60:40) film was cut on the surface and healed by heating at 75 °C for 10 min.^21^ A solvent mixing method was used to blend graphene nanoplatelets with PVAc in chloroform, followed by drying in a silicone mold and hot pressing into composite sheets.^22^ The effect of self-healing on the scratches on this PVAc/graphene nanocomposite film was demonstrated by using an optical microscope. The self-healing ability of the film was attributed to the intrinsic shape memory behavior of PVAc and the diffusion of the PVAc chains on the damaged surfaces after heating above their glass transition temperature (*T*_g_).^22^

Cellulose nanomaterials (CNMs) have been embedded in PVAc to obtain composites with different mechanical and viscoelastic properties.^23−30^ Sisal derived cellulose nanocrystals (CNCs) embedded in a PVAc matrix (at concentrations of 0, 1.0, 2.5, 5.0, and 10 wt %) have been demonstrated to hinder the diffusion of water in the composite before the nanocrystal content increased to the percolation threshold.^23^ Rusli et al. reported that the local orientation of tunicate cellulose whiskers (now more commonly called CNCs), mixed with a PVAc matrix, influenced the stress-transfer inside the composites:^25^ CNCs in solution-cast samples were found to be isotropic, and a lower level of molecular deformation was detected using Raman spectroscopy in these regions compared with regions where they were uniaxially orientated in compression molded samples.^25^ Gong et al. reinforced PVAc with 10 wt % cellulose nanofibrils (CNF) using a twin-screw extruder (TSE), which enhanced the tensile strength and modulus, storage modulus, and the creep elasticity and viscosity of the material.^26^ Adding 1 wt % of CNMs to PVAc has been reported to improve the elastic stiffness and shear strength of spruce and beech joints glued with a CNM/PVAc composite adhesive, most significantly compared to samples with 0.5 and 2 wt % CNMs.^30^

As the mechanical properties of composites using CNMs as reinforcement are likely affected by the dispersion of the nanomaterials and their interfacial adhesion with a polymeric matrix, much research has aimed to tune the mechanical properties of the composites by altering the surface properties of CNMs.^24,27,28^ PVAc latex composite films were fabricated by mixing CNFs (5, 10, 20, and 30 wt %) with aqueous suspensions of PVAc particles with poly(vinyl alcohol) (PVA) as a stabilizer, in which the nanofibrils influenced the viscoelastic properties of the film.^24^ The interactions between the CNFs and the hydrophilic PVA in the matrix contributed to an increase in the storage modulus.^24^ Glycidyl methacrylate (GMA) was grafted to both a CNC reinforcement and a polylactide (PLA) matrix to enhance their interfacial adhesion.^27^ PVAc has also been premixed with GMA-grafted CNCs, improving tensile properties and the thermal resistance of a composite.^27^ Sapkota et al. compared solution-cast CNC/PVAc specimens with samples processed with roller blade mixers (RBMs) or TSEs after solution casting.^28^ This study showed that postprocessing CNC/PVAc composites with TSE resulted in lower mechanical properties compared with the original samples, while the RBMs made no difference.^28^

As the above examples show, cellulose nanomaterials (CNMs) have primarily been used as fillers in PVAc matrices without forming any covalent or ionic bonds with the polymer.^23−30^ The self-healing functionality of cellulose- and PVAc-based composites has not been studied thus far. CNMs are typically incorporated into PVAc through mechanical methods, such as solvent mixing and extrusion, with no reported surface modification of CNMs to improve their compatibility with PVAc. Additionally, the ATRP of PVAc using cellulose nanocrystals (CNCs) as macroinitiators has yet to be reported. In the present work, CNCs were modified by esterification followed by activators generated by electron transfer (AGET) ATRP of vinyl acetate, grafting PVAc onto the CNCs. The grafted CNCs were then fabricated into self-healing films after mixing with bulk PVAc.

## Materials and Experimental Methods

### Materials

Extra pure ethylene glycol 99+% was purchased from Thermo Fisher Scientific (Lancashire, UK). Potassium periodate 99.8%, *N*,*N*-dimethylformamide (DMF), and 3-amino-1-propanol were purchased from Merck Life Science UK Ltd. (Dorset, UK). Freeze-dried CNCs (sodium form) with a 0.94 wt % sulfur content were purchased from the Process Development Center, University of Maine (Maine, USA). l-Ascorbic acid was purchased from Fisher Scientific UK. α-Bromoisobutyryl bromide (BIBB), copper(I) bromide, 4-dimethylamino pyridine (DMAP), *N,N,N*′*,N*″*,N*″ -pentamethyl diethylenetriamine (PMDTA), triethylamine (TEA), and polyvinyl acetate (*M*_w_ ≈ 100,000 g mol^–1^) were purchased from Merck Life Science UK Limited. Vinyl acetate was purchased from Alfa Aesar Avocado Research Chemicals, Ltd.

### Synthesis of Aminopropanol CNC (apCNC)

To graft amino propanol groups onto sCNCs, CNCs were first oxidized using periodate into dialdehyde CNCs (DACNCs) following a similar procedure described in previously published articles.^31,32^ Briefly, a sCNC suspension (3 wt %) was reacted with 1.68 mmol of sodium periodate per 1 g of CNCs for 48 h at room temperature, followed by washing with DI water and dialysis against DI water with a cellulose membrane (molecular weight cutoff of 14 kDa) over 48 h. No glycol quenching was used for the washing of DACNCs, which has been reported to be unnecessary and likely to induce contaminations and changes in the chemical structure of the DACNCs.^33^ The DACNC suspension then underwent a reductive amination by reacting first with 3-aminopropanol at 45 °C and pH 4–5 for 3 h, followed by the addition of sodium cyanoborohydride reducing agent and reaction at room temperature in the dark for 24 h. The samples were centrifuged and washed with DI water three times and finally dialyzed against DI water to remove excess reactants.

### Synthesis of CNC Macroinitiator (BrCNC)

The fabrication procedure for the CNC macroinitiator was adjusted according to a method described in a previous publication.^34^ Freeze-dried CNCs (500 mg) were first dispersed in dimethylformamide (DMF) (50 mL) and then placed in an ice bath. DMAP (2 g) was then added to this suspension while stirring. The mixture was deoxygenated with a Schlenk line three times, and reactants triethylamine (TEA) and α-bromoisobutyryl bromide (BIBB) were added dropwise (4 mL of each). After a 24 h period, the reaction was stopped by exposing it to air and adding ethanol (EtOH), and the CNCs were washed and centrifuged with acetone and deionized water. Acetone was used for the centrifugation, and DI water was used for the dialysis for 6 days. At the end of this process, a dried BrCNC powder was obtained by freeze-drying.

### AGET ATRP on BrCNC

The AGET ATRP of VAc was conducted in DMF. BrCNCs (1 mmol) were suspended in DMF (20 mL) and stirred overnight at 45 °C. PMDETA, CuBr, ascorbic acid (AA), and vinyl acetate (VAc) were added to the suspension under a nitrogen atmosphere in the proportions listed in Table S1. The reaction was conducted at a temperature of 70 °C for 24–48 h. The reaction was terminated on exposure to air and cooling. Then, CNCs reacted with VAc (VAcCNC) were washed with DI water, filtered out with glass microfiber filters, and dried in a vacuum oven at 50 °C for 12 h. VAcCNC_200_ and VAcCNC_1000_ were obtained, named according to the molar ratio between VAc and CNC as shown in Table S1.

### Fabrication of PVAc-VAcCNC Composite Films

PVAc was dissolved in acetone at 10% (w/v) at room temperature with stirring for 20 min. The PVAc-acetone solution was subsequently added dropwise onto the VAcCNC_1000_ sample, which had been filtered and dried in the previous steps, maintaining a PVAc to VAcCNC weight ratio of 1:10. After the evaporation of acetone at room temperature for about 5 min, the mixture was pressed into a film and dried for 24 h under ambient conditions. An additional and comparative sample was made with sCNCs and PVAc-acetone solutions using the same methodology but with the PVAc:sCNC ratio increased from 1:10 to 5:10 to make a consolidated composite film.

### Fourier Transform Infrared (FTIR) Spectroscopy

The sCNCs, apCNCs, BrCNCs, and VAc-CNCs samples were dried before being characterized and compared using FTIR spectroscopy with a Spectrum 100, PerkinElmer, USA, system. The modified CNCs were ground into powders prior to testing. FTIR was performed in transmission mode with 4 scans from 4000 to 400 cm^–1^ for each sample with the resolution set to 2 cm^–1^. The absorbances located in the range 4000 to 400 cm^–1^ for each sample were normalized with a band located at ∼1030 cm^–1^ after performing a baseline correction to the data.

### ^1^H Solution-State Nuclear Magnetic Resonance (NMR) Spectroscopy

During the AGET ATRP process, 0.3 mL of the reacting mixture was removed using a syringe needle after reacting at time points of 0, 3, 9, and 24 h. The reaction solvents were filtered and mixed with 0.5 mL of DMSO-*d*_6_, and a ^1^H spectrum (Jeol ECZ400 NMR) was acquired at 20 °C, 64 scans, 400 MHz, and a recycle delay of 4 s. The peak area integration between VAc (CH at 4.56 ppm) and the solvent DMF (CH at 7.92 ppm) was calculated for each spectrum to monitor the residual amount of monomer in the reacting mixture. Comparative experiments were carried out where sCNCs replaced BrCNCs and were mixed with VAc under similar AGET ATRP conditions. The change in the VAc concentration in these comparative experiments was also monitored using ^1^H solution-state NMR.

### X-ray Photoelectron Spectroscopy (XPS)

XPS was performed using an Argus spectrometer and a monochromatic Al Kα (1486.7 eV) X-ray source. Powder-form sCNC and BrCNC samples were pressed into high-purity indium foils (99.9975%, Goodfellow) that were scraped clean prior to analysis. A charge neutralizer (operating at a beam energy of 4.5 eV and an electron flux of 3 μA) was used to mitigate sample charging built up during the X-ray exposure. Owing to the possibility of beam damage to the Br bonding environment, Br scans were performed first. Wide scans were measured using a 50 eV pass energy, while narrow high-resolution scans were conducted with a 20 eV pass energy. High-resolution scans for carbon, oxygen, and bromine were treated using a Shirley-type background subtraction. All energy scales were charge-referenced to the C–O peak of cellulose located at 286.4 eV. C 1s, O 1s, N 1s, S 2p, and Br 3d core levels were used for quantification, and the photoionization cross sections of Scofield were used with TNY angular distribution parameters.^35,36^

### Transmission Electron Microscopy (TEM)

For TEM images of dried VAcCNC suspensions (around 1 mg/mL), the CNCs were dried and ground before being dispersed in DI water ultrasonically with a sonic probe (Branson Digital Sonifier). The suspensions were drop-cast onto carbon-coated grids and stained with a 2% uranyl acetate solution. A JEM-2100F TEM (200 kV field emission gun) from JEOL, Japan, was used to image the dried VAcCNCs on the grid. Healed CNC-PVAc samples were embedded in Durcupan resin mixed in a silicone rubber mold. The resin was cured at a temperature of 60 °C in an oven for 48 h. Sections of embedded copolymer were cut to a thickness of 80 nm on a ultramicrotome Ultracut E. The cut thin sections were imaged in a Talos L120C (Thermofisher UK) transmission electron microscope.

### Solid-State NMR

^1^H–^13^C cross-polarization magic angle spinning (CP/MAS) NMR was performed on a Bruker Avance III NMR spectrometer operating at a ^1^H frequency of 300.13 and 100.21 MHz for ^13^C. The samples were packed into an 80 mL zirconia rotor and spun at an MAS rate of 10 kHz. All ssNMR spectra were acquired using a ^1^H π/2 *rf* pulse of 3.2 μs, a contact time of 2 ms, and a recycle delay of 10 s at a temperature of 20 °C. Global spectral deconvolution was carried out using the MestreLab MNova (v14.2) package.

### Thermogravimetric Analysis (TGA) and Differential Scanning Calorimetry (DSC)

sCNCs, BrCNCs, and VAcCNCs were tested in an STA Instruments (NETZSCH STA 449 F3 Jupiter) under nitrogen, within a temperature range of 30–600 °C at a heating rate of 10 °C min^–1^. Derivative thermogravimetric curves were obtained by performing a first derivative on the percentage weight loss data from the TGA data. DSC (DSC 2500, TA Instruments, Waters Corporation, UK) was performed on the VAcCNC sample, reacted with different ratios of VAc (VAcCNC_200_, VAcCNC_1000_), and the composite films were fabricated with PVAc and VAcCNC_1000_. The samples were heated from 20 to 120 °C at a rate of 5 °C min^–1^, and then cooled to −40 °C at −5 °C min^–1^. These samples were subsequently reheated to 120 °C under the same heating rate. sCNCs and pure commercial PVAc were tested under the same conditions. Pinholes were made into each crucible lid to allow water vapor or solvent residues to escape during the heating process.

### Scanning Electron Microscopy (SEM) and Energy Dispersive X-ray (EDX) Analysis

A JSM-IT300 (JEOL, Japan) system was used to obtain all of the SEM images and carry out the EDX analysis. For CNCs and PVAc-VAcCNC samples, a high-resolution sputter coater from Afar Scientific, UK, was used to coat silver onto the samples and the sample holders. Secondary electron detector (SED) images were captured by using an accelerating voltage of 15 kV with a working distance of 50 mm under a high vacuum mode.

### Self-Healing Test on Composite Films

Both sCNC and VAcCNC_1000_ composite films were cut into two pieces by using a scalpel. The separated pieces were placed together (two halves in close proximity) on aluminum foil. They were then placed in an oven for healing at a temperature of 40 °C for 6 h. The healed sample was broken into small pieces, and a fragment was then tested using a microindenter (FT-MTA03, FemtoTools AG, Switzerland) and a spherical probe (diameter 47.7 μm). Array tests, consisting of indents made in 20 rows with 10 measurements on each row (red dots in Figure S5), were conducted on the healed film, with one row of indents located on the “healed line”. The maximum force for the tests was set to be 1800 μN. The stiffness measured on the healed line was compared to measurements within the nondamaged areas. All the samples were viewed before and after healing using an optical microscope to visualize the healing effect. The stiffnesses of the pure PVAc film and sCNC-PVAc film were also measured with the microindenter. A Hertz model^37^ was used to obtain the Young's modulus from indentation curves of VAcCNC-PVAc and sCNC-PVAc films, whereas the approach proposed by Oliver et al.^38^ was used to take the observed plastic deformation into account when calculating the Young's modulus of the pure PVAc film.

## Results and Discussion

### Characterization of the BrCNC Macroinitiators

**Scheme 1:**
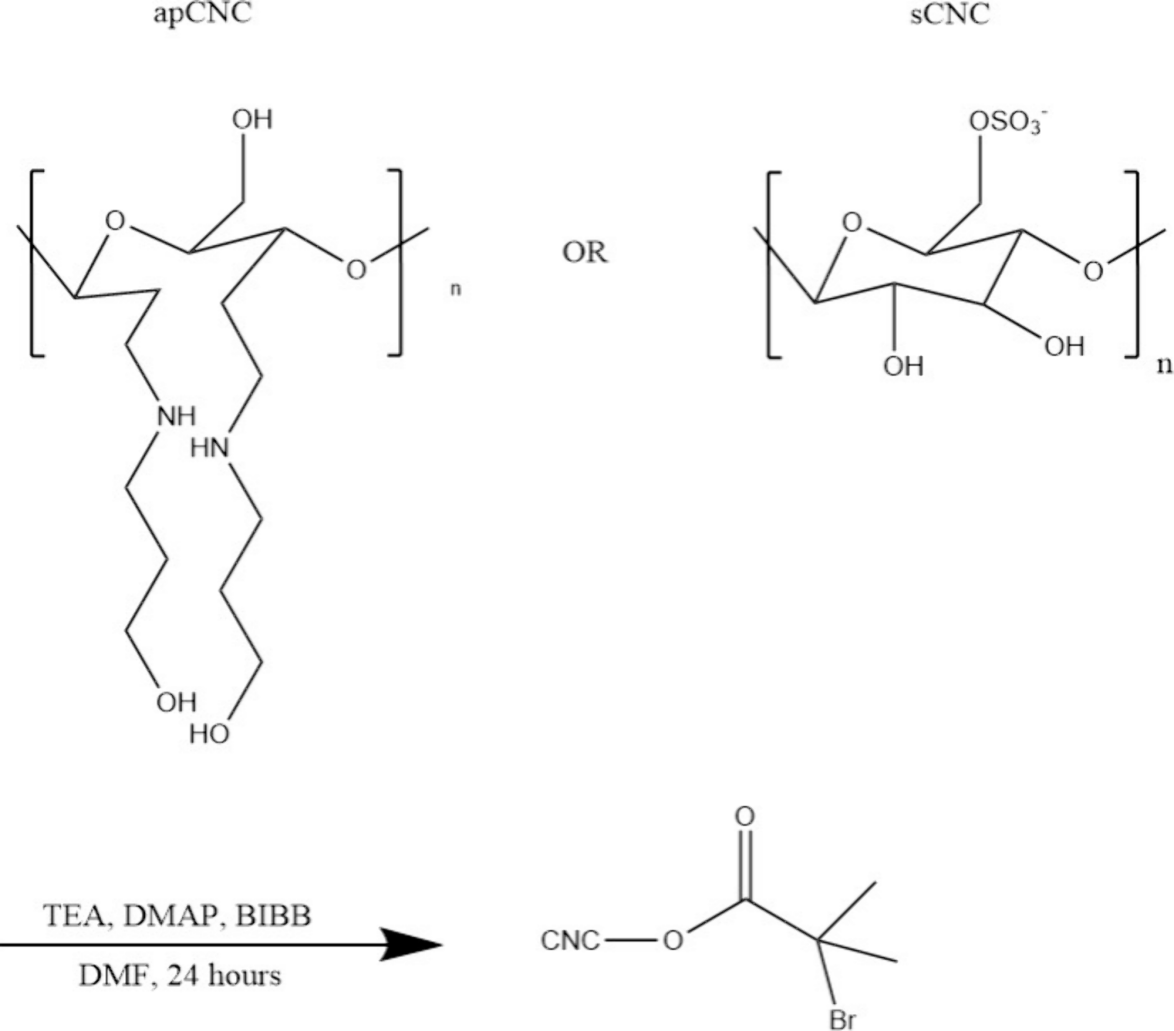
Esterification of apCNC and sCNC into a Macroinitiator BrCNC with Triethylamine (TEA), 4-Dimethylamino Pyridine (DMAP), and α-Bromoisobutyryl Bromide (BIBB) in Dimethylformamide (DMF)

Two types of CNC macroinitiators were fabricated by an esterification reaction occurring on the hydroxy groups of CNCs (Scheme 1). CNCs grafted with aminopropanol groups (apCNCs) on C2 and C3 by reductive amination were compared with sCNCs (as supplied) to investigate the regioselectivity of the esterification reaction. sCNCs are thought to have hydroxy groups at C2, C3, and C6, while apCNCs have hydroxy groups at C6, and two aminopropanols on C2 and C3 (Scheme 1).

**Figure 1:**
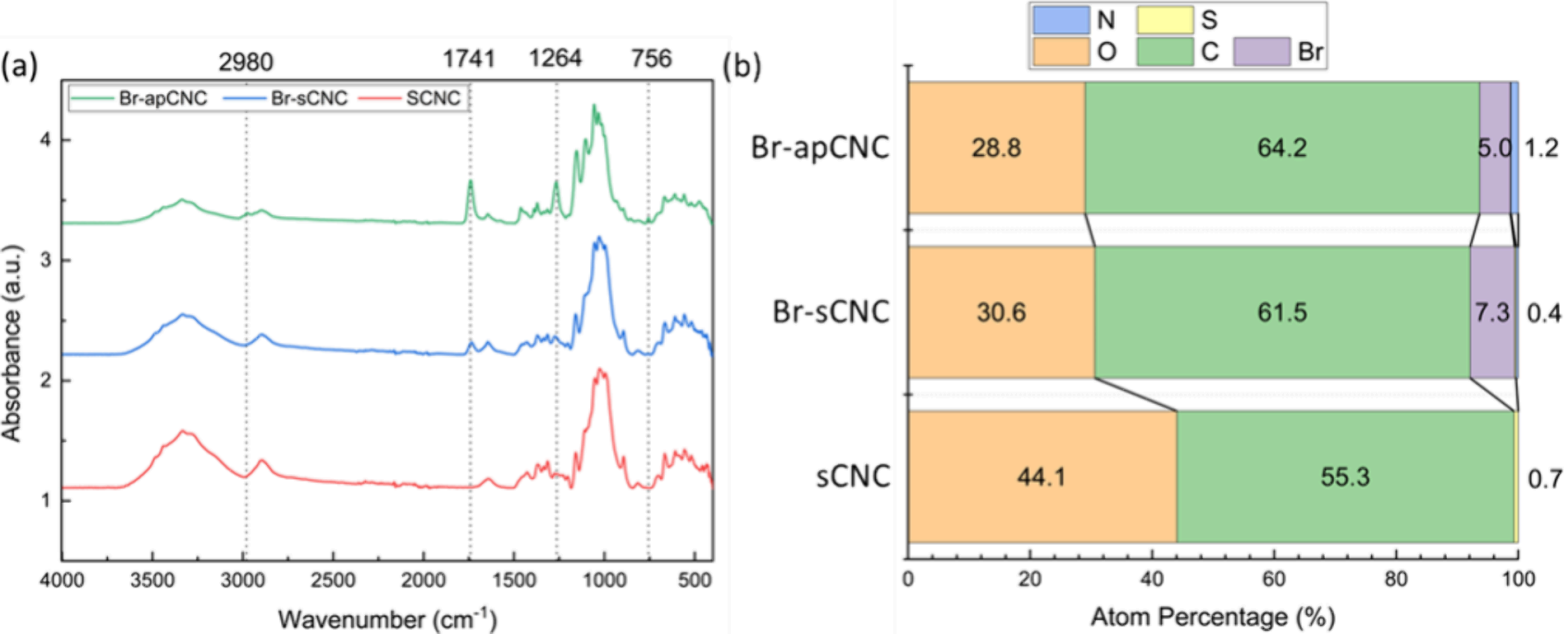
(a) Typical FTIR absorbance spectra for sCNC, Br-apCNC and Br-sCNC. a.u. – arbitrary units. Dotted lines indicate the positions of the bands located at ∼756, ∼1264, ∼1741, and ∼2980 cm^–1^ (b) Elemental analysis of CNCs by X-ray photoelectron spectroscopy (XPS). Values are reported as percentage quantities (atom%). These results have been estimated based on tabulated theoretical photoelectron cross sections.

In the characterization of CNC macroinitiators, FTIR spectra (Figure 1a) confirmed the presence of grafted ester groups on the CNCs, while XPS analysis provided insights into their elemental composition (Figure 1b). The IR absorption of C=O stretching in the grafted ester group was observed at a wavenumber position of 1741 cm^–1^ for BrCNCs, which was not present for sCNCs. The C–O stretching for a grafted ester group at 1264 cm^–1^ was present for both brominated Br-apCNC and Br-sCNCs. The presence of C=O and C–O stretch bands for the ester groups demonstrated that esterification had successfully occurred. A stronger absorbance intensity of both groups (C=O and C–O) in Br-apCNCs compared with Br-sCNCs possibly indicated that more ester groups were present on Br-apCNCs due to the presence of more reaction sites (OH). The appearance of additional bands located at 756 and 2980 cm^–1^, corresponding to C–H stretching modes, in the Br-apCNC spectrum, confirms the grafting of carbon chains onto the CNCs. The successful esterification was further proven by XPS and solid-state NMR.

XPS was also used to detect elements within the macroinitiators and compare with sCNC as a control sample (Figure 1b). The accuracy of the tabulated theoretical photoelectron cross sections is not high, however, the precision of the measurements is good, meaning differences between samples can be reliably estimated. The lower percentage of bromine on the Br-apCNCs sample (5.0% compared to 7.3% on the Br-sCNC sample) might be caused by the additional carbon chains and amine groups associated with the aminopropanol, increasing the amount of carbon and nitrogen and their percentage in the overall quantification. The carbon content for Br-sCNC was higher than that for sCNC, caused by the grafted carbons present through esterification. A nitrogen content of 1.2% was detected for Br-apCNCs, which is expected to be introduced through the reductive amination process and is higher than the levels found for sCNCs and Br-sCNCs. Br-apCNCs, compared to Br-sCNCs, with additional carbon chains grafted with the ester groups and a similar proportion of the Br element, were used for the AGET ATRP of vinyl acetate.

### Characterization of the CNC-PVAc Copolymer

**Scheme 2:**
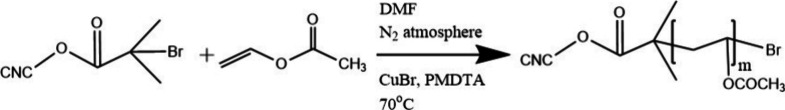
ATRP Reaction Pathway of Polyvinyl Acetate Graft from CNC Macroinitiators

AGET ATRP conducted on the Br-apCNC is shown in Scheme 2, with Br-apCNCs noted as BrCNCs in subsequent discussions. ^1^H NMR spectroscopy was used to monitor the consumption of monomers in the reaction solution (shown in Tables S2–S4 and Figures S1–S3, Supporting Information). Polymerized PVAc-CNC copolymers are labeled VAcCNC_200_ and VAcCNC_1000_ according to the monomer and macroinitiator ratio (Table S1).

**Figure 2:**
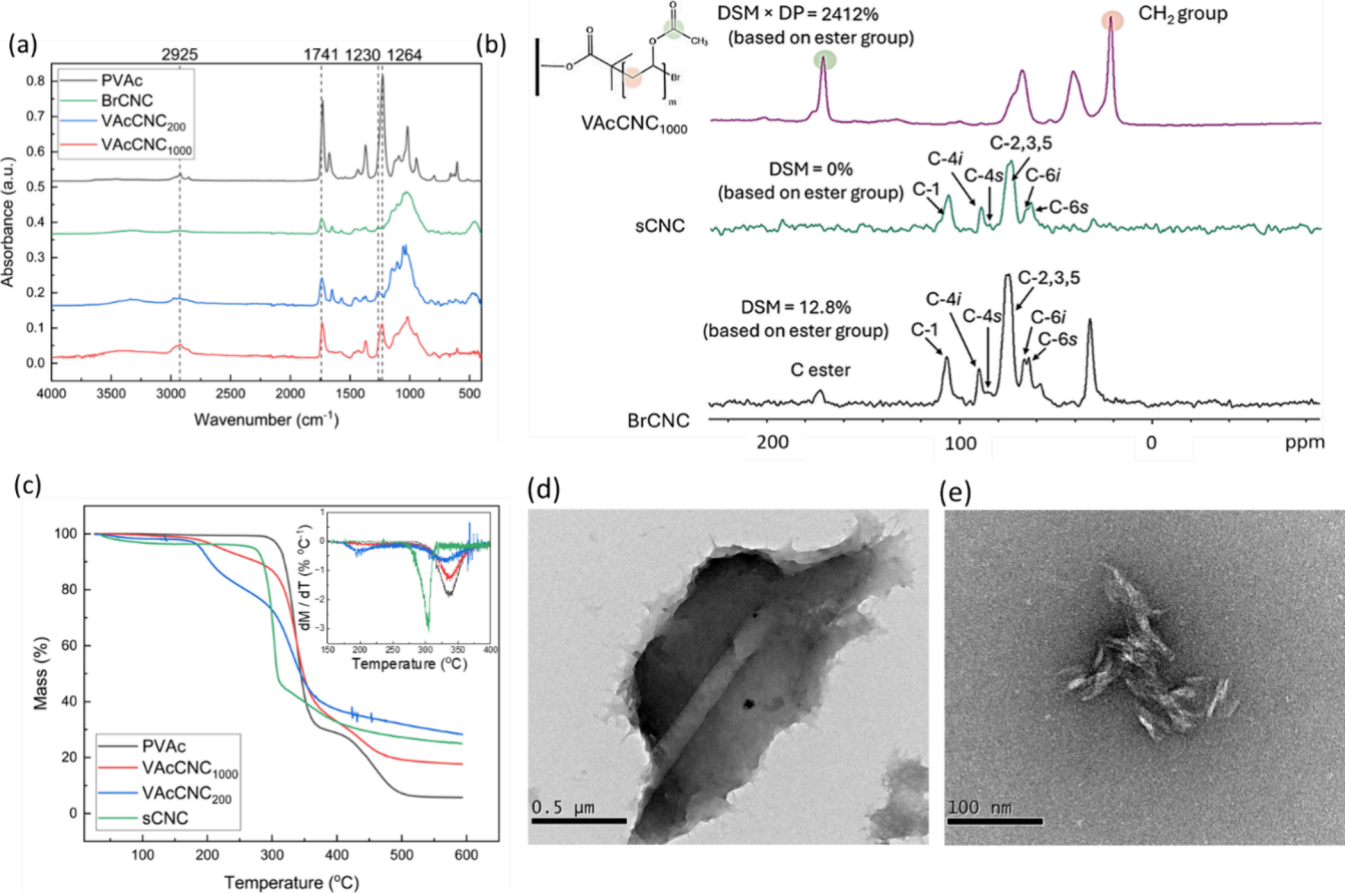
(a) Typical FTIR spectra for BrCNC, VAcCNC_200_, VAcCNC_1000_, and commercial PVAc, a.u. – arbitrary units, (b) ^1^H–^13^C CP MAS NMR spectra of sCNC, BrCNC and VAcCNC_1000_ with the degree of surface modification (DSM) and degree of polymerization (DP) estimated from the NMR results; (c) thermogravimetric analysis (TGA) and derivative data (inset) for sCNC, VAcCNCs, and PVAc in nitrogen; (d) TEM images of filtered and (e) centrifuged VAcCNC_1000_ samples dispersed in water. Note: the large impression left in the center of the material in (d) is due to the microfiber filter and is not solid material.

FTIR was used to characterize the functional groups on CNCs before and after the polymerization reaction. As shown in Figure 2a, BrCNCs exhibit bands located at 1741 and 1264 cm^–1^, corresponding to C=O and C–O stretching in the ester groups, and a band located at 2980 cm^–1^, corresponding to the −CH stretch mode in the carbon chain. In contrast, the C–O stretching mode on VAcCNC_1000_ and pure PVAc appeared at 1230 cm^–1^, and the −CH stretching in the carbon chain appeared at 2925 cm^–1^. A clear shift in these two stretching modes is thought to be induced by the grafting of PVAc chains by the ATRP reaction. An increase in the intensity of bands located at 1230, 1741, and 2925 cm^–1^ from BrCNC to VAcCNC_200_ and VAcCNC_1000_ likely indicates an increase in the number of repeating VAc groups grafted onto the CNCs’ surfaces.

The ^1^H–^13^C CP/MAS NMR spectra of sCNC, BrCNC, and VAcCNC_1000_ (Figure 2b) demonstrated the presence of ester groups on the BrCNC surfaces and on the repeating units of PVAc chains on VAcCNC_1000_. The degree of surface modification (DSM) of BrCNC was calculated to be 12.8% for BrCNC according to eq 1,
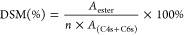
1where *A*_ester_ is the area under the deconvoluted −COO– peak (*ca*. 173 ppm) of the sample, and A_(C4s+C6s)_ is the area under the deconvoluted peaks associated with the cellulose backbone surface moieties of C4 and C6 carbons (*ca*. 85 and 63 ppm, respectively). The *n* parameter accounts for the number of carbons in the ester moiety, which is 1 for BrCNC, and is equal to the average number of repeating vinyl acetate groups in each PVAc chain (degree of polymerization, DP) for VAcCNCs after polymerization. As the ATRP reaction only occurs on the Br groups on the BrCNCs, the DSM of the VAcCNC_1000_ was assumed to be the same as that of the BrCNCs (eq 2). The DP of the PVAc groups on VAcCNC_1000_ was then calculated to be 188 according to eq 3.
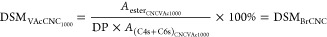
2
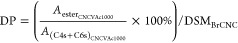
3

TGA was used to follow the decomposition behavior of the CNC-PVAc copolymers and compared to sCNC and PVAc (Figure 2c). The onset degradation temperature for VAcCNCs was found to be located at ∼200 °C, which is about 100 and 130 °C lower than those of sCNCs and PVAc, respectively. For the VAcCNCs, a second decomposition occurred at ∼330 °C, which is at the same temperature as the PVAc decomposition. This second decomposition was not observed for the sCNC. This indicates that the decomposition of CNCs and the grafted PVAc chains in VAcCNCs occur at different temperatures. TEM images of VAcCNCs obtained from two different approaches using filtration and centrifugation are shown in Figure 2d,e. The filtered VAcCNCs were found in a film form, and the glass microfiber filter left a fiber-like indentation on the film (see Figure 2d). At the edge of the film, a separation of multiple layers was also observed. As for the samples washed by centrifugation, CNC clusters were found in the TEM images (Figure 2e). These clusters seem to aggregate strongly enough to withstand ultrasonic treatment, with no isolated CNCs observed in the suspension, which is thought to be mainly driven by the entanglement of polymer chains grafted by ATRP.^39^ The samples obtained from filtration (Figure S4, Supporting Information) were subsequently used to produce self-healing composite films, with the VAcCNC_1000_ working as the matrix of the composite. VAcCNC_1000_ formed free-standing films after filtration and drying (Figure S4); however, the modified CNC film itself has not shown any self-healing behavior.

### Self-Healing Composite Film Characterization

**Figure 3:**
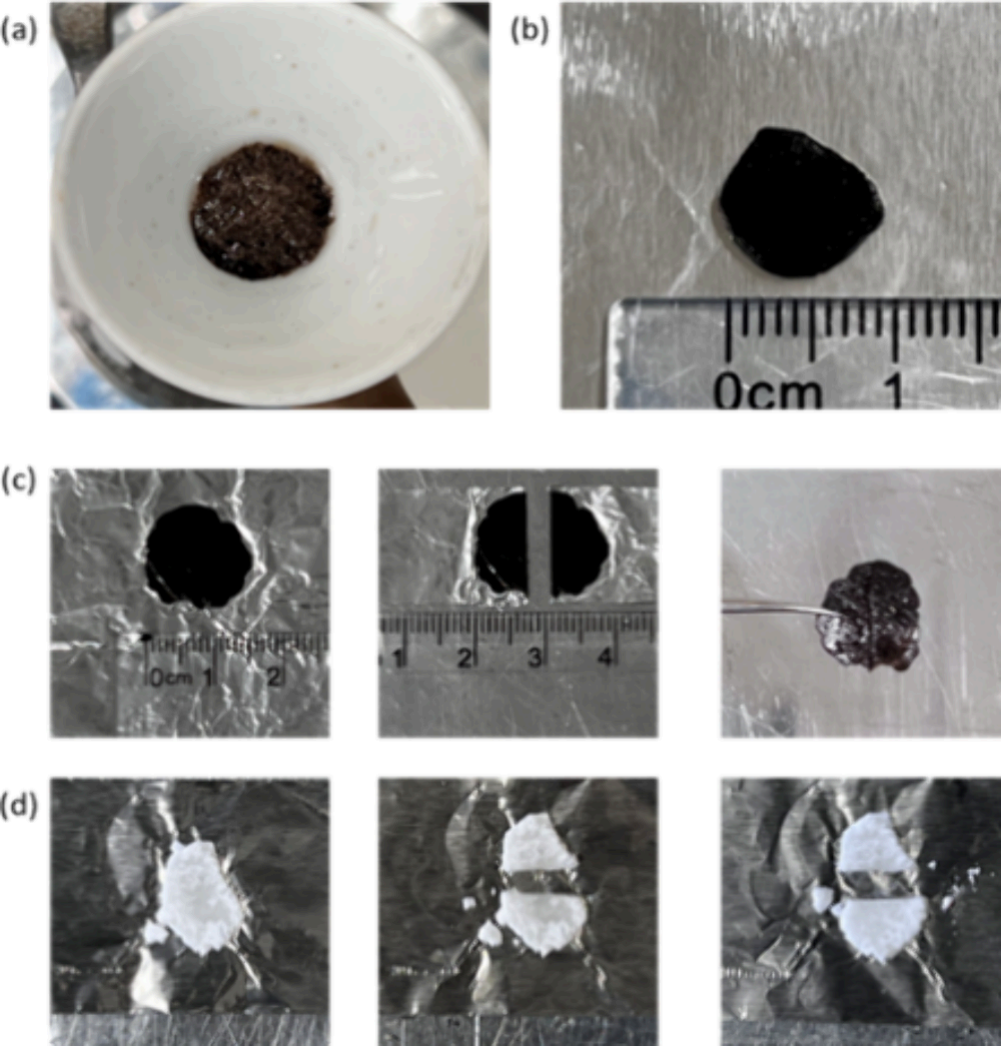
Photographs of (a) Filtered VAcCNC_1000_, (b) VAcCNC_1000_ mixed with 10% PVAc, (c) compressed 10% PVAc-VAcCNC film cut and healed at 40 °C for 3 h, and (d) compressed 50% PVAc-sCNC film cut and heated at 40 °C for 3 h.

PVAc-VAcCNC composite films (PVAc:VAcCNC = 1:10 wt/wt) were fabricated by adding dropwise a PVAc/acetone solution onto the filtered VAcCNC film, evaporating the acetone and applying compression (Figure 3a,b).

In contrast, when mixed with 10% PVAc, sCNCs failed to form an integrated film and remained as separated CNC clusters. The fraction of PVAc in sCNC needed to be increased to 5:10 to enable sufficient PVAc chains to obtain a free-standing sCNC composite film. A self-healing test was done for the bulk PVAc film at 40 °C prior to a self-healing test on composite films. This confirmed that the PVAc chain mobility at 40 °C was efficient for self-healing with enough chain entanglement forming at the contact fracture surfaces (Figure S6, Supporting Information). Both VAcCNC and sCNC composite films were cut into two pieces, rejoined, and secured by compression while heating to 40 °C in an oven. As shown in Figure 3c,d, after 3 h of heating, the black PVAc-VAcCNC film healed into a single, cohesive piece that could be easily held with tweezers, whereas the white PVAc-sCNC film remained separated. A clear mark remained visible on the healed film after 3 h of heating. To further heal the sample and completely mitigate the cut, it was heated for a further 3 h and placed under an optical microscope for imaging. Microindentation of the sample was also performed for mechanical characterization.

**Figure 4:**
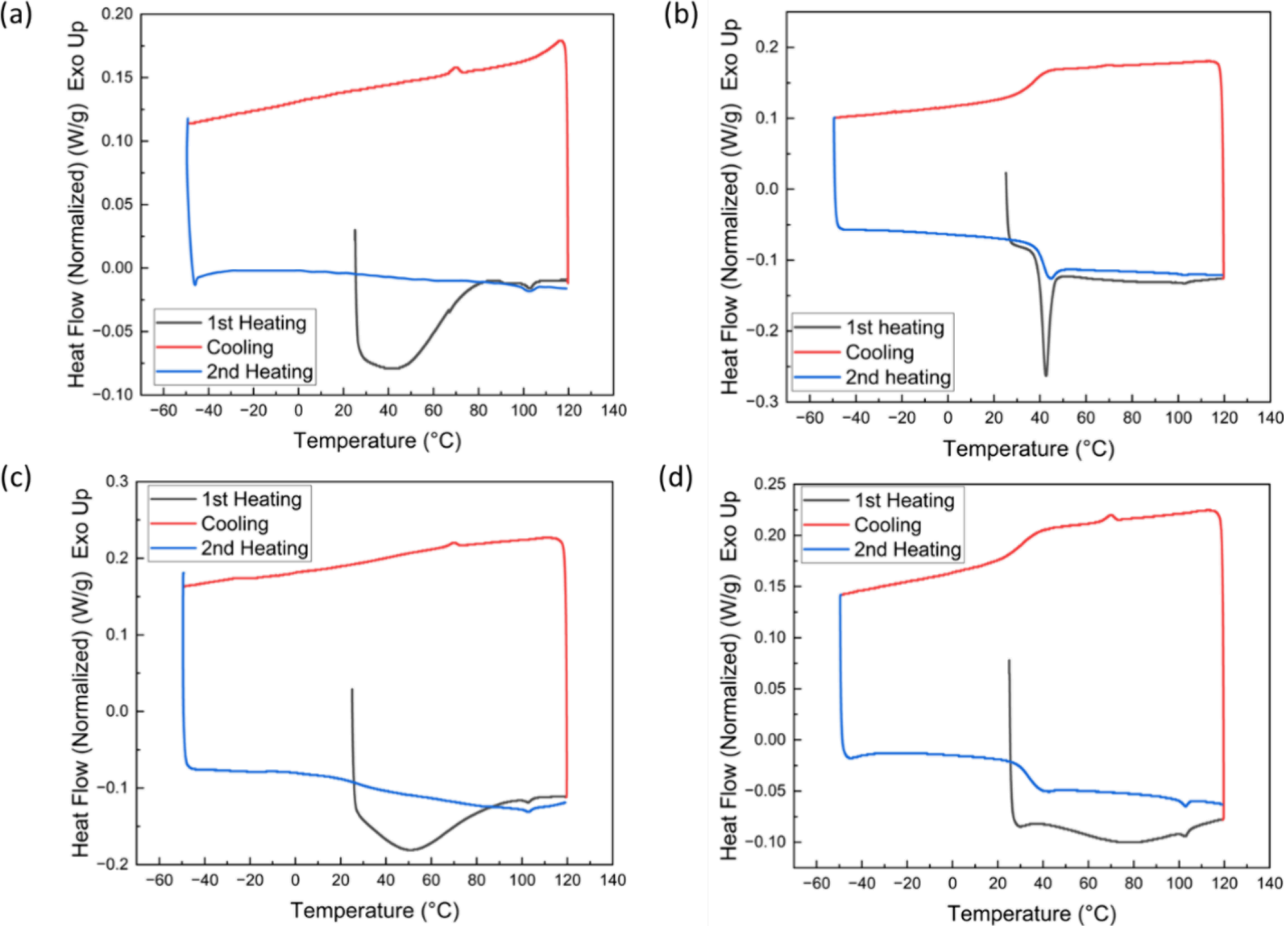
Differential scanning calorimetry (DSC) heat–cool–heat cycle data for (a) sCNC, (b) bulk PVAc, (c) filtered VAcCNC_1000_, and (d) VAcCNC_1000_ mixed with 10% PVAc.

The healing temperature was chosen to be 40 °C according to the DSC results of the 10% PVAc-VAcCNC film, PVAc polymer, sCNC, and VAcCNC_1000_. For all the CNC samples (Figure 4a,c), an endothermic peak (39–50 °C) appeared in the first heating cycle within the DSC profile, corroborating other studies.^40,41^ The enthalpy for this endothermic peak is thought to be associated with the evaporation of bound water in the CNC samples. The peak disappeared during the second heating cycle as the pinhole in the lid allowed the water vapor to escape. The second heating curve for sCNC from −20 to 100 °C is smooth, with no visible peaks. A glass transition-like change occurred for the VAcCNC_1000_ sample at 30 °C, likely due to the mobility of PVAc chains. Clear melting peaks occurred at 43 and 45 °C separately in the first and second round of heating cycles for the bulk PVAc sample (Figure 4b). A glass transition was noted in the second heating cycle for PVAc prior to the melting peak with the midpoint of the transition at 41 °C.

The DSC curve of the VAcCNC_1000_-PVAc composite sample combined the features appearing in the individual curves for VAcCNC_1000_ and PVAc (Figure 4b–d). In the first heating cycle, an endothermic peak related to water evaporation was prominent, while this same peak moved to a higher temperature (76 °C) compared with those for sCNCs and VAcCNC_1000_. This shift in the peak position might have been caused by the additional PVAc chains hindering the water from escaping. A glass transition appeared in the second heating cycle at 34 °C, between those of VAcCNC_1000_ (30 °C) and pure PVAc (41 °C). Similar to bulk PVAc, a small melting peak appeared in Figure 4d after the glass transition at a temperature of 40 °C.

**Figure 5:**
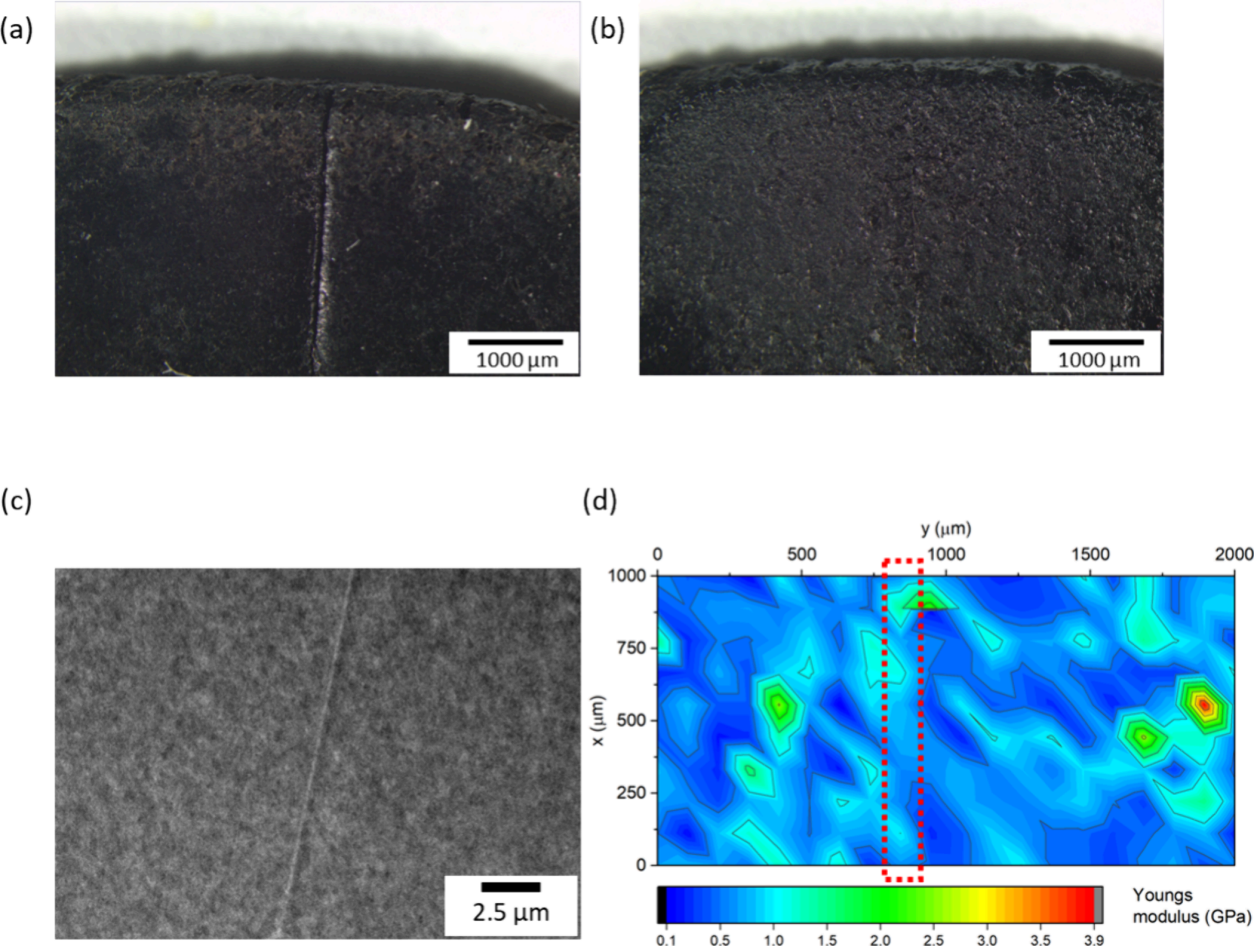
Typical optical microscope image of (a) 10% PVAc-VAcCNC film before healing, (b) 10% PVAc-VAcCNC film after healing for 6 h at 40 °C, (c) TEM image of a cut section of healed VAcCNC-PVAc composite film, and (d) Young’s modulus map obtained from the microindentation array tests (20 × 10) (Figure S5) providing a detailed representation of the mechanical properties across the surface of the healed composite film. The area highlighted by a dotted red rectangle in (d) shows the region where the sample was healed.

The optical microscope images shown in Figure 5a,b illustrate how the films healed after having been heated to 40 °C for 6 h. The cut on the 10% PVAc-VAcCNC film was barely visible under the optical microscope after healing. Although the PVAc-sCNC film showed a glass transition and melting point in the DSC heating curves (Figure S7 Supporting Information), it had no signs of healing under the same healing process, and the cut remained unchanged (Figure S8a,b, Supporting Information). A TEM image of a cut thin section of the healed sample showed the detailed morphology of the healed region inside the composite film and the inner structure of the fractured surfaces (Figure 5c). The surface of the sCNC-PVAc composite films was rough, as observed from the SEM image (Figure S8d, Supporting Information), while the surface and the edge of the VAcCNC composite film seemed to be smoother (Figure S8d, Supporting Information). It is thought that the sCNCs at the fracture surface lack interactions with PVAc, leading to poor interfacial adhesion, while the two fracture surfaces are packed closely, hindering PVAc chain entanglement and effective healing.

Compared to the PVAc/PCL^21^ and PVAc/graphene^22^ self-healing composites, the VAcCNC-based system offers sustainability (CNCs from renewable resources) and enhanced interfacial adhesion between CNCs and PVAc due to covalent grafting. The VAcCNC-PVAc system showed self-healing at 40 °C, which is lower than the previously reported healing temperatures (Table 1). The proportion of bulk PVAc polymers added to VAcCNCs was also much lower than that used in previous research (Table 1). The VAcCNC-PVAc composites utilized VAcCNC as the matrix material with bulk PVAc serving as the filler, whereas the PVAc-graphene and PVAc-PCL system employed the opposite configuration.

**Table 1:** Comparison of the Thermal Characteristics of PVAc-Based Self-Healing Composites

system	healing temperature (°C)	melting point of the other component (°C)	PVAc: other component (w/w)
PVAc/PCL^21^	75	PCL: 60	60:40
PVAc/graphene^22^	60	graphene: no	95.5:4.5
VAcCNC/PVAc	40	VAcCNC: no	10:100

Microindentation array data yielded a map of stiffness over the healed CNC-PVAc film surface (Figure 5d). With an average Young’s modulus of 0.72 ± 0.44 GPa, the healed region (red rectangle) exhibited the same stiffness as the intact areas. Young’s modulus for a pure PVAc film was reported to be 0.3 GPa in a recent study^42^ and was measured to be 0.25 ± 0.13 GPa from the microindentation test on our sample. The measured stiffness of the sCNC-PVAc (PVAc: sCNC = 50:100) sample (0.72 ± 0.39 GPa) was similar to the stiffness of the VAcCNC-PVAc (PVAc:VAcCNC = 10:100) sample.

No change in the surface stiffness in the healed region on the VAcCNC-PVAc sample was observed after healing in the oven at 40 °C for 6 h, which indicates that the material has fully reformed. The self-healing capability of the VAcCNC-PVAc film is thought to be attributed to the diffusion and entanglement of PVAc chains grafted on the CNCs at the damaged surfaces when heated above their glass transition temperature (Tg). These grafted PVAc chains exhibit a lower degree of polymerization and a reduced Tg compared with bulk PVAc, indicating that they are more flexible and will readily form entanglements with other PVAc chains at elevated temperatures.

## Conclusions

A self-healing composite film of modified CNCs combined with a commodity thermoplastic material has been demonstrated. CNCs with poly(vinyl acetate) chains polymerized on their surface were combined into these films with 10% bulk PVAc. Sulfated (sCNCs) and aminopropanol (apCNCs) CNCs were compared in an esterification reaction. It was found from XPS studies that by grafting aminopropanol groups on C2 and C3, the proportion of Br on the surface of Br-sCNCs and Br-apCNCs are similar, while the Br-apCNCs have additional carbon chains. After poly(vinyl acetate) chains were grafted using the Br-CNC macroinitiators, the resulting VAcCNCs were found to form closely packed clusters, which are thought to be caused by the entanglement of grafted PVAc chains. These polymer chains grafted on CNCs decomposed at a higher temperature compared to those on the sCNCs. The VAcCNCs were found to be more compatible with PVAc compared with sCNCs, owing to the presence of PVAc chains, which allowed the preparation of a composite with a uniform structure. The VAcCNC-PVAc composite films exhibited a self-healing ability at 40 °C due to the entanglement of PVAc chains on the VAcCNCs with bulk PVAc at the fracture surfaces. Complete recovery of the film surface stiffness was demonstrated by using microindentation mapping. The healed area demonstrated the same stiffness compared to that of the intact areas. The fabrication method of the self-healing PVAc-CNC composite film introduced an approach for compounding CNCs with polymers and holds potential for designing other functional composites. Thermoplastic-based composite materials are one approach to sustainability in that they allow for the molding and repair of structures. Our approach described herein could be one way to achieve this capability.

## Supporting Information Available

^1^H solution-state NMR, sample photographs, microindentation, and SEM (PDF)

## Abbreviations

AGET ATRP: activators generated by electron transfer atom transfer radical polymerization,
ATRP: atom transfer radical polymerization
BIBB: 2-bromoisobutyryl bromide
BrCNC: brominated cellulose nanocrystal
CNC: cellulose nanocrystal
CNM: cellulose nanomaterial
CRP: controlled/living radical polymerization
DACNC: dialdehyde cellulose nanocrystal
DETA: diethylenetriamine
DI water: deionized water
DMAP: 4-dimethylamino pyridine
DMF: dimethylformamide
DSM: degree of surface modification
FTIR: Fourier transform infrared spectroscopy
GMA: glycidyl methacrylate
PCL: poly(ε-caprolactone)
PMDTA: *N*,*N*,*N*′,*N*″,*N*′′-pentamethyl diethylenetriamine
PVA: poly(vinyl alcohol)
PVAc: polyvinyl acetate
RAFT: reversible addition–fragmentation transfer
RBM: roller blade mixer
ROP: ring-opening polymerization
sCNC: sulfated cellulose nanocrystal
SEM: scanning electron microscopy
SSNMR: solid-state nuclear magnetic resonance
STA: simultaneous thermogravimetric analysis
TEA: triethylamine
TEM: transmission electron microscopy
TSE: twin-screw extruder
TGA: thermogravimetric analysis
VAcCNC: vinyl acetate modified cellulose nanocrystal
XPS: X-ray photoelectron spectroscopy
