# Supplementary material for: A Self-Healing Composite Film Made of Cellulose Nanocrystals and a Polyvinyl Acetate Copolymer
Source: ACS Appl Polym Mater. 2025 Apr 16;7(8):4982–91. doi: 10.1021/acsapm.5c00219 (PMC12038788; doi:10.1021/acsapm.5c00219)
Supplement: Supplementary file 1 — ap5c00219_si_001.pdf [file ap5c00219_si_001.pdf]

Supporting Information

A self-healing composite film made of cellulose nanocrystals and a polyvinyl acetate copolymer

*Guofan Xu,[1] Jude Laverock,[2] Todor T. Koev,[3] Yaroslav Z. Khimyak,[3] Onajite Abafe Diejomaoh,[1] Sebastien Rochat [2,4] and Stephen J. Eichhorn,[*1]*

1. Bristol Composites Institute, School of Civil, Aerospace and Design Engineering , University of Bristol, University Walk, Bristol, BS8 1TR, UK.

2. School of Chemistry, University of Bristol, Bristol, BS8 1TS, UK.

3. School of Chemistry, Pharmacy and Pharmacology, University of East Anglia, Norwich Research Park, Norwich, NR4 7TJ, UK.

4. School of Engineering Mathematics and Technology, University of Bristol, Bristol, BS8 1TW, UK.

Corresponding Author

* Stephen J. Eichhorn, s.j.eichhorn@bristol.ac.uk

A bulk PVAc (Mw ~ 100,000 g mol^{-1}) film was made by dissolving PVAc beads in acetone, casting and drying for 48 hours under ambient conditions (Figure S6a). The PVAc film with diameter ~1 cm and thickness 0.8±0.02 mm was then cut into two pieces and healed in oven at 40˚C for 3 hours (Figure S6b&c).

The DSC curve for the 50% PVAc-sCNC sample (Figure S7) combined the features appearing in the individua curves of sCNC and PVAc (Figure 4a&b). In the first heating cycle, an endothermic peak at 76 ˚C related to moisture residue evaporation in sCNC got combined with the melting point of PVAc at 46 ˚C. In the second cycle of heating, the melting point of PVAc appeared at 45 ˚C with a clear glass transition showed at 41 ˚C which was same as the 2nd heating cycle of the pure PVAc sample (Figure 4b).

EDX analysis was carried out in the same regions captured by SEM to obtain the local compositions of the films (Figure S7c&d). Disregarding the Ag element, which is from the conductive coating, 73.0% of C, 24.4% of O and 0.1% of Br were detected on the VAcCNC sample, while 62.7% of C, 35.6% of O and 0.18% of S were detected on the sCNC sample. No Br was detected on the sCNC composite films while the concentration of S was too low in VAcCNC composite films to be detected, and no Cu residue was detected on the VAcCNC samples. The black colour of the VAcCNC itself, and the resulting composite sample, is thought to derive from the presence of Br on the CNCs.

Table S1 Reactants proportion and experimental conditions for ATRP of polyvinyl acetate.

	BrCNC / mmol	PMDETA : CuBr: Vinyl acetate : AA	Temp/ °C	Time /h
$VAcCNC_{200}$	1	0.2:0.2:200:0.2	70	24
$VAcCNC_{1000}$	1	0.2:0.2:1000:0.4	70	48

Table S2 ^{1}H NMR peak integrations changing over time of vinyl acetate-sCNC in DMF solution under ATRP reaction conditions.

Time /h	0.0	3.0	6.0	9.0	24.0	48.0
DMF CH peak integration at 7.92 ppm	1.13	1.14	1.14	1.15	1.16	1.18
Vinyl acetate CH peak integration at 4.56 ppm	0.87	0.86	0.86	0.85	0.84	0.82
Monomer : solvent ratio	0.77 : 1	0.76 : 1	0.75 : 1	0.74 : 1	0.72 : 1	0.69 : 1
Monomer residue percentage /%	100	98	98	96	94	90

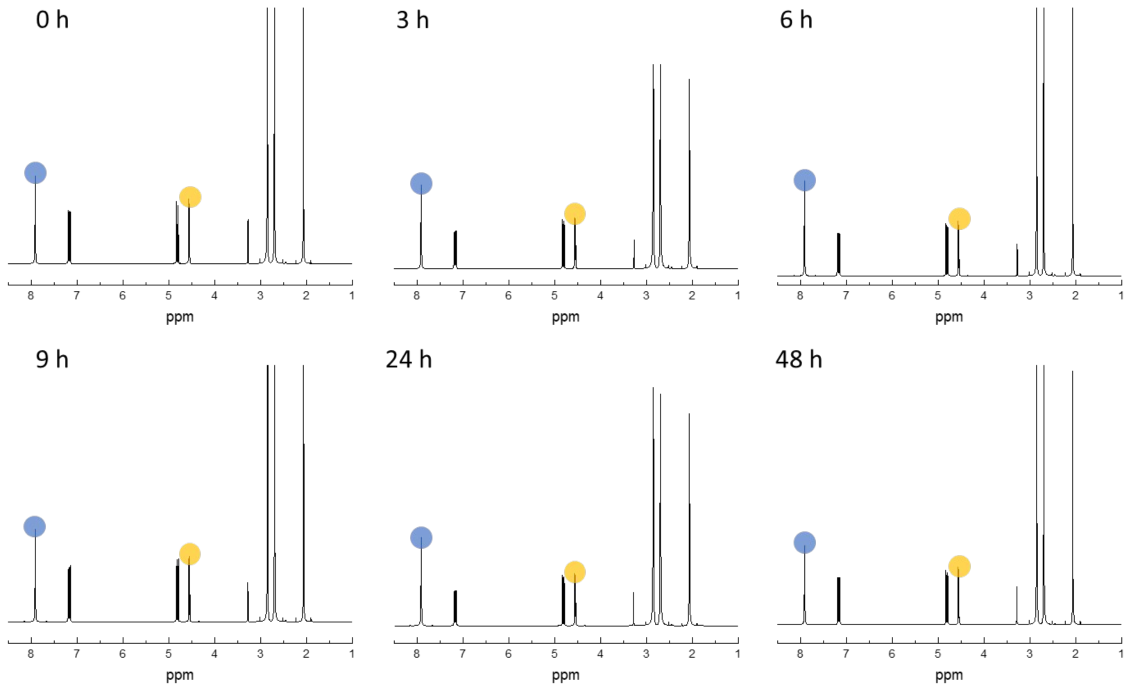

Figure S1 1H NMR spectra of vinyl acetate in DMF solution in DMSO-d6, with the peak of vinyl acetate CH (4.56 ppm) circled in yellow and the peak of DMF CH (7.92 ppm) circled in blue.

Table S3 1H NMR peak integrations changing over time of vinyl acetate : BrCNC 200 : 1 DMF solution under ATRP reaction conditions.

Time /h	0.0	3.0	9.0	24.0
DMF CH peak integration at 7.92 ppm	1.18	1.99	4.98	1.00
Vinyl acetate CH peak integration at 4.56 ppm	0.82	1.01	1.02	0
Monomer : solvent ratio	0.69 : 1	0.51 : 1	0.20 : 1	0 : 1
Monomer residue percentage /%	100	73	29	0

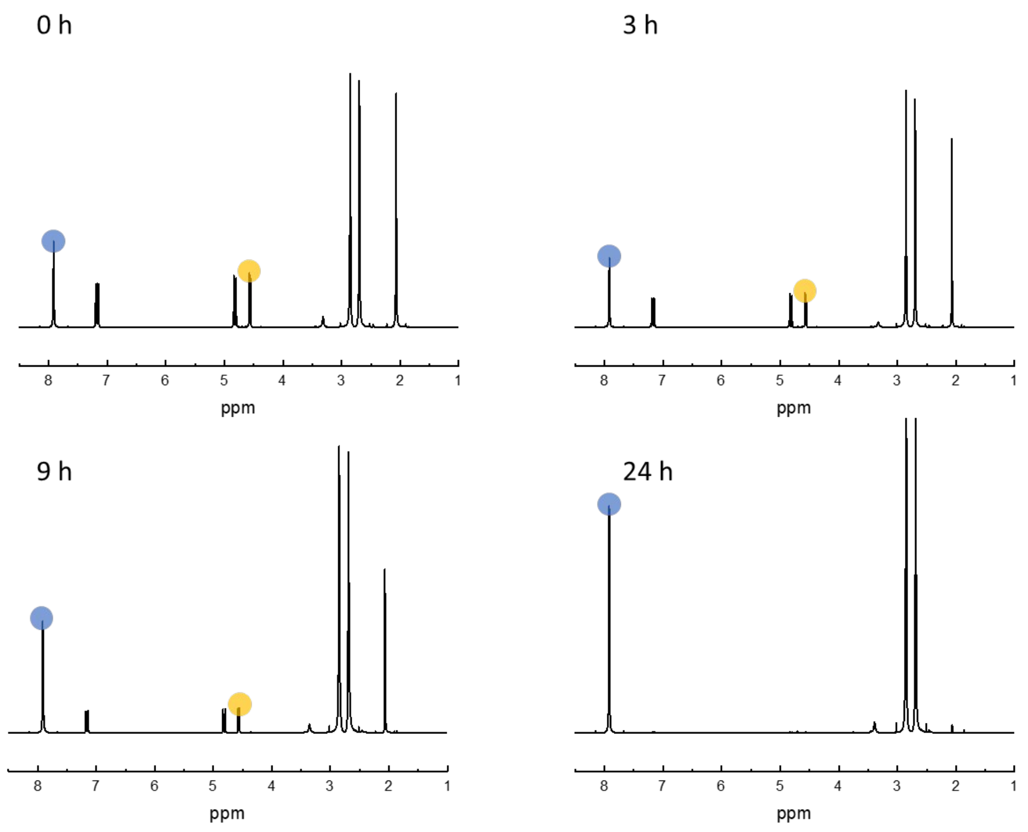

Figure S2 1H NMR spectra of vinyl acetate : BrCNC 200 : 1 DMF solution in DMSO-d6 , with the peak of vinyl acetate CH (4.56 ppm) circled in yellow and the peak of DMF CH (7.92 ppm) circled in blue.

Table S4 1H NMR peak integrations changing over time of vinyl acetate : BrCNC 1000 : 1 DMF solution under ATRP reaction conditions.

Time /h	0.0	3.0	24.0	48.0
DMF CH peak integration at 7.92 ppm	1.1	1.13	2.09	1.00
Vinyl acetate CH peak integration at 4.56 ppm	0.9	0.87	0.91	0
Monomer : solvent ratio	0.82 : 1	0.77 : 1	0.43 : 1	0 : 1
Monomer residue percentage /%	100	94	53	0

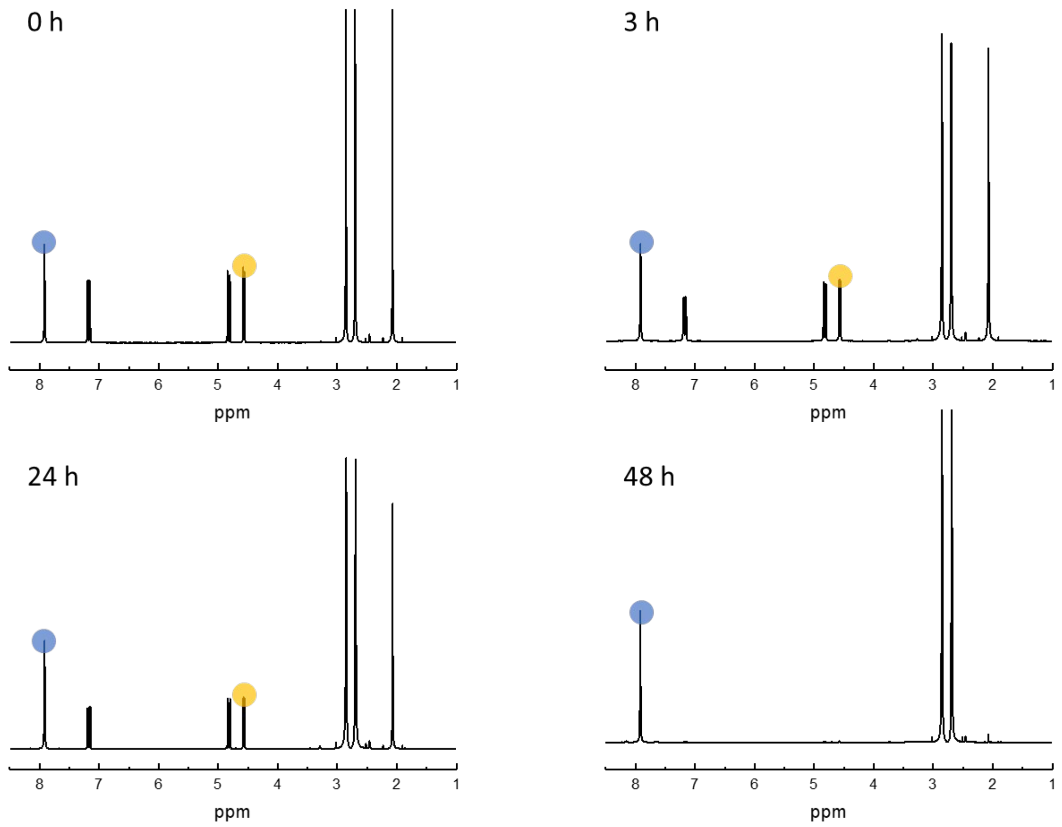

Figure S3 1H NMR spectra of vinyl acetate : BrCNC 1000 : 1 DMF solution in DMSO-d6 , with the peak of vinyl acetate CH (4.56 ppm) circled in yellow and the peak of DMF CH (7.92 ppm) circled in blue.

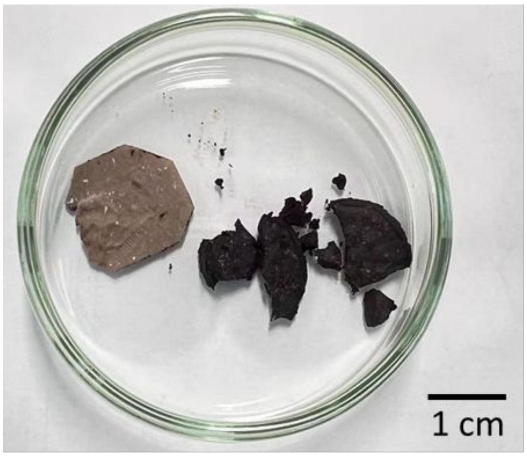

Figure S4 A photograph of dried VAcCNC copolymer and glass microfibre filter.

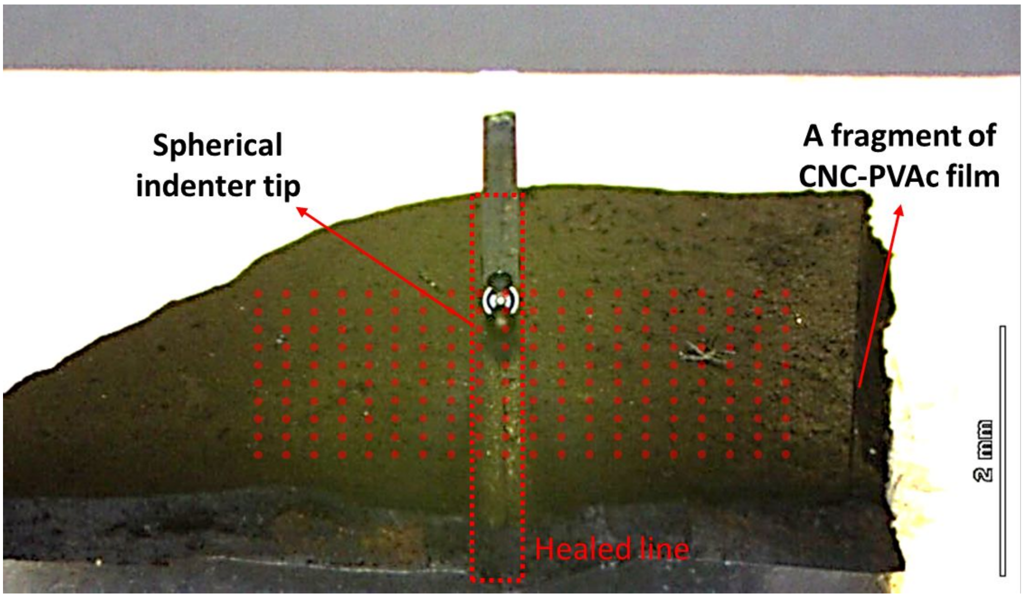

Figure S5 Array tests (20 × 10) of microindentations on a fragment of healed composite film.

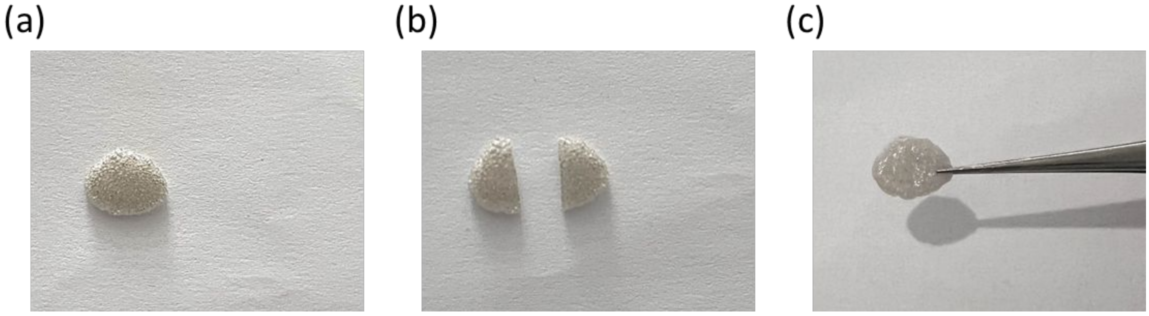

Figure S6 Typical photos of (a) bulk PVAc film, (b) the same PVAc film cut in two pieces and (c) healed PVAc film by heating at 40 °C for 3 hours.

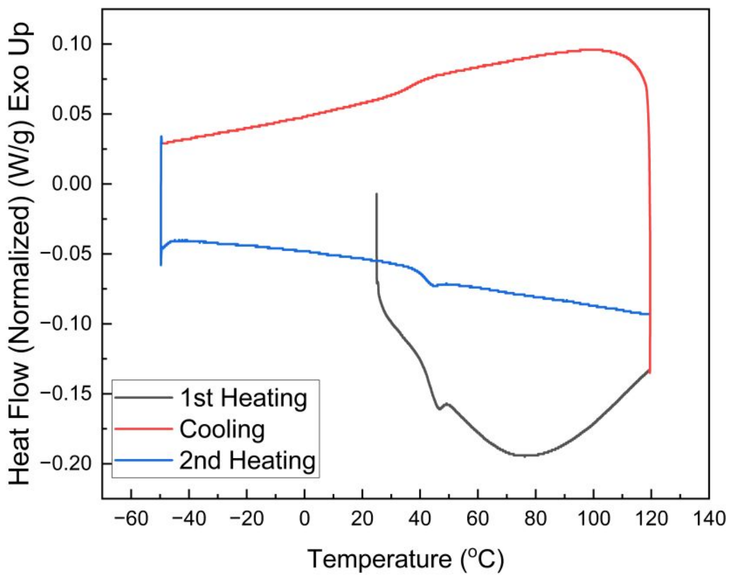

Figure S7 Differential scanning calorimetry (DSC) heat-cool-heat cycle data for 50 : 100 PVAc-sCNC sample.

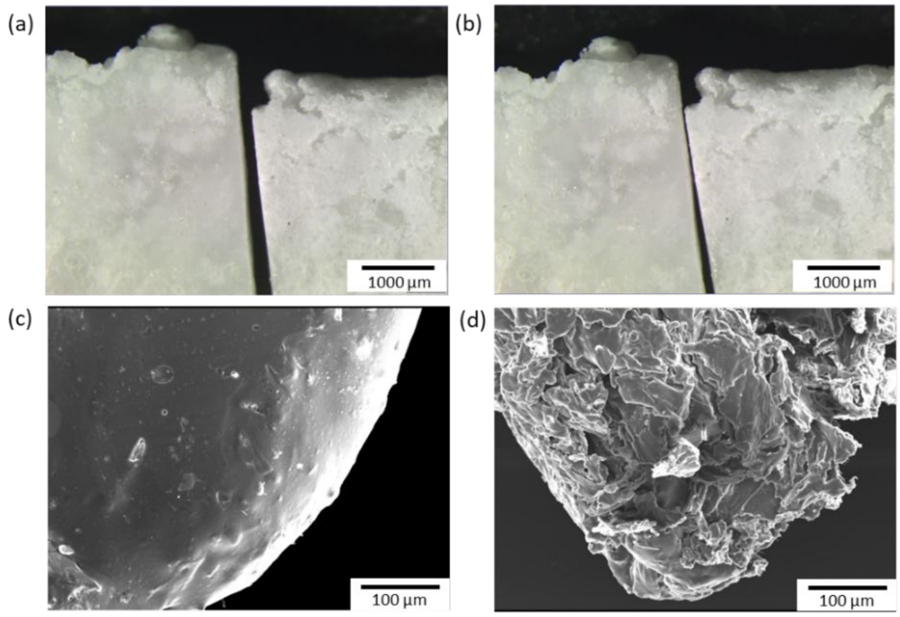

Figure S8 Typical optical microscope image of (a) 50% PVAc-sCNC film before healing, (b) 50% PVAc-sCNC film after heating at 40 °C for 6 hours and secondary electron SEM images of (c) 10% PVAc-VAcCNC and (d) 50% PVAc-sCNC.
